# Uterine tumors resembling ovarian sex cord tumors: A retrospective analysis of 7 cases from a single institution

**DOI:** 10.1097/MD.0000000000038824

**Published:** 2024-07-05

**Authors:** Zhe Ma, Ying Li

**Affiliations:** a Department of Gynecology and Obstetrics, Tianjin Amcare Women’s and Children’s Hospital, Tianjin, China; b Department of Gynecology and Obstetrics, Tianjin Central Hospital of Gynecology and Obstetrics, Tianjin, China

**Keywords:** clinic, diagnosis, pathology, prognosis, sex cord markers, Uterine tumors resembling ovarian sex cord tumors

## Abstract

To investigate the clinicopathological features, diagnosis, surgical treatment and prognosis of uterine tumors similar to ovarian sex cord tumors (UTROSCT). The clinical data, surgical approach, histopathological, and immunohistochemical features of 7 cases of UTROSCTs were retrospectively reviewed and followed up. All 4 patients were premenopausal women. The most common clinical presentation was menorrhagia (n = 4) followed by postmenopausal lower abdominal mass (n = 2) and postmenopausal bleeding (n = 1). Gynecological ultrasonography suggested uterine fibroids in 4 cases, adenomyosis with uterine fibroids in 2 cases, and an intrauterine mass in 1 case. Pelvic MRI was performed preoperatively in only 2 cases, and both indicated uterine fibroid degeneration, including 1 patient with suspected malignancy. Preoperative serum tumor markers were measured in 6 patients, and only 1 patient had elevated CA125 levels, up to 158 U/mL. Total hysterectomy with bilateral adnexectomy or salpingectomy was the most common treatment pattern (n = 6). The tumors were located within the myometrium (n = 4), submucosa (n = 1), and isthmus to external cervical os (n = 1), with a range of 2 to 12 (mean = 8) cm. Edema and degeneration were observed in 2 cases, and necrosis in 1 case. Postoperative follow-up ranged from 31 to 82 (mean = 43) months. Unfortunately, 1 patient died at 54 months of follow-up without undergoing hysterectomy. The remaining 6 cases showed no tumor recurrence or metastasis after surgery. Histological examination revealed a tumor composed of epithelioid tumor-like cells arranged in cords, trabeculae, and nests. All 7 tumors showed expression of 2 sex cord differentiation markers. Furthermore, all tumors expressed the smooth muscle marker, while epithelial marker CK (4/7). endometrial stromal marker CD10(0/7). The Ki-67 proliferation index was found to be <5% (5/7). The option of total hysterectomy may be considered for women who do not have any fertility requirements. However, for young women who desire to maintain their reproductive capacity, surgery to preserve the uterus may be an alternative, although it necessitates careful postoperative monitoring. In terms of follow-up monitoring, MRI is more suitable than ultrasound. The diagnosis of UTROSCT heavily relies on histopathological examination and immunohistochemical analysis.

## 1. Introduction

Uterine tumors resembling ovarian sex cord tumors (UTROSCT) are rare gynecological tumors. Scully and Clement were the first to report 14 uterine tumors in 1976.^[1]^ They classified uterine tumors with sex cord differentiation into 2 types: type Ι (6 of 14) exhibited endometrial stromal tumors with a sex cord-like component, with endometrial stromal component dominating and generally comprising 10% to 40% of the tumor, and type ΙΙ (8 of 14) displayed a predominantly or wholly epithelioid component with no distinct endometrial stroma, hence referred to as a uterine tumor resembling an ovarian sex cord tumor. And they defined type ΙΙ tumors as UTROSCT for these neoplasms. Now, this terminology is only used for the type ΙΙ tumors in study. According to the fifth edition of the World Health Organization (WHO) classification of tumors of the female reproductive organs, UTROSCT belongs to the category of uterine heterogeneous mesenchymal tumors.^[2]^

Due to the clinical rarity of UTROSCT and the prevalence of case reports in recent years, the rate of preoperative diagnosis is low. It is typically diagnosed following a hysterectomy or lumpectomy. The aim of this study was to collect clinical data, surgical procedures, pathological features, immunohistochemical findings, and prognostic data from 7 tumors. Additionally, relevant literature was reviewed to enhance our understanding. This study emphasizes the importance of early diagnosis in UTROSCT, as it enables the development of personalized treatment plans based on each patient condition, ultimately leading to improved prognostic outcomes.

## 2. Materials and methods

General clinicopathological data was collected for 7 cases of UTROSCT diagnosed at Tianjin Central Hospital of Gynecology and Obstetrics from January 2016 to December 2020. Of the 7 cases, 6 patients underwent transabdominal/trans-laparoscopic total hysterectomy with bilateral adnexal/bilateral salpingectomy, 1 patient which was considered to be malignant, performed pelvic lymph node resection. One patient only underwent diagnostic curettage. Clinical (patient age, symptoms at time of presentation), gross features, pathological and immunohistochemical features were obtained from the medical records, and the cutoff for telephone follow-up was April 2023.

Tumor sample tissues were collected and fixed in a 4% neutral formaldehyde solution. They were then dehydrated in graded alcohols, embedded in paraffin, and cut into 4 μm thick serial sections. The sections were observed under a microscope after Hematoxylin and Eosin staining (HE) were staining. For immunohistochemical staining, corresponding wax blocks were selected and the envision method was used. The antibodies used for detection included: CD56, CD99, Calretinin, WT-1, α-Inhibin, Desmin, Caldesmon, vimentin, SMA, CK, CD10, Ki67, CGA, syn, CD34, CD117, and Melan-A. All tissue sections were reviewed by 2 or more highly qualified pathologists.

## 3. Results

### 3.1. Clinical features

The clinicopathological data of the 7 UTROSCT cases are shown in Table 1. The patients ranged from 38 to 66 (median = 52; mean = 52.9) years. Three patients were postmenopausal, while 4 were not. Clinical presentation was known in 7 patients, menorrhagia being most common (n = 4) followed by postmenopausal lower abdominal mass (n = 2) and postmenopausal bleeding (n = 1). Examination revealed concomitant anemia in 5 patients, with 2 of them having severe anemia (HB:51 g/L and 57 g/L). Ancillary examinations, including gynecological ultrasound, indicated uterine fibroids in 4 out of 7 patients (57.14%), adenomyosis combined with uterine fibroids in 2 (28.57%), and an intrauterine mass in one (14.29%). Only 2 cases underwent pelvic MRI examination before operation, all of which suggested degeneration of uterine fibroids, including 1 case of suspected malignant tumor. Preoperative serum tumor marker tests were conducted on 6 patients, including CA125, CA199, CEA, AFP, and HE4. Only 1 patient presented with an elevated CA125 marker of up to 158 U/mL.

**Table 1:** Clinicopathological data of 7 cases of UTROSCT.

Case	Age at diagnosis (yr)	Postmenopausal	Symptoms	Anemia	Comorbidities	Tumor location	Size of lesion (cm)	Therapy at diagnosis	Follow-up (mo)	Status
1	52	No	Bleeding	Yes	Adenomyosis Uterine fibroids	/	/	D&C	54	Death: The cause was not determined
2	53	Yes	Asymptomatic	No	Adenomyosis Uterine fibroids	Intermuscular	11	LTH + BSO	82	NED
3	63	Yes	Asymptomatic	Yes	None	Intermuscular	12	TAH + BSO	39	NED
4	49	No	Bleeding	Yes	None	Isthmus to the external orifice of the cervix os	6	LTH + BSO	31	NED
5	49	No	Bleeding	Yes	Adenomyosis Uterine fibroids	Intermuscular	10	LTH + BS	31	NED
6	66	Yes	Bleeding	No	None	Submucosal	5	TAH + BSO + LN	33	NED
7	38	No	Bleeding	Yes	Adenomyosis	Intermuscular	4	TCRM + LTH + BS	31	NED

BS = bilateral salpingectomy, BSO = bilateral salpingo-ovariectomy, D&C = diagnostic curettage, LTH = laparoscopic total hysterectomy, NED = no signs of disease, TAH = abdominal total hysterectomy, TCRM = hysteroscopic myomectomy.

One patient only underwent diagnostic curettage without surgical treatment. The remaining 6 patients were treated by transabdominal/laparoscopic total hysterectomy with bilateral adnexectomy/ salpingectomy. Among them 1 patient, who was 38 years and underwent hysteroscopic resection of an intrauterine mass, was diagnosed with UTROSCT. Because she had no fertility requirements, she then underwent laparoscopic total hysterectomy and bilateral salpingectomy. The postoperative pathology revealed no tumor residue in the myometrial wall. One patient, whose pelvic MRI suspected malignant tumor, performed pelvic lymph node resection. Postoperative pathological pelvic lymph nodes were not found metastasis (0/34).

### 3.2. Pathological features

**Figure 1.**
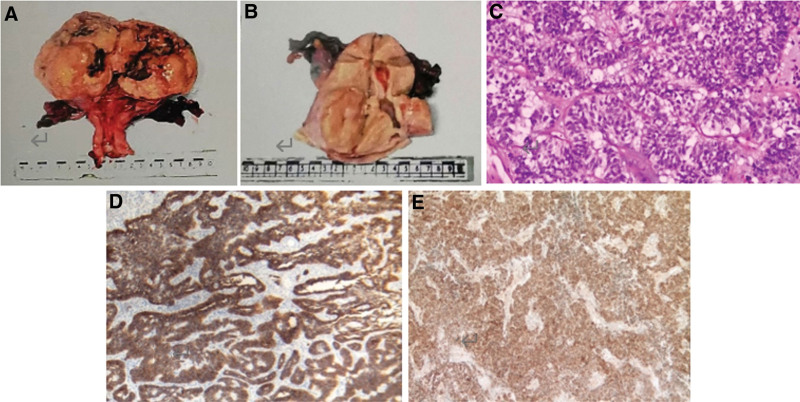
(A) Section grayish yellow soft, slightly fine, edema, hemorrhage and necrosis. The cystic cavity contains a significant amount of yellow-brown clear fluid. The inner wall is smooth, with a localized granular protrusion, and clearly demarcated from the muscle wall. (B) The intramuscular mass extends from the isthmus toward the outer portion of the cervix. The boundary is well-defined, and there is easy local exfoliation. The deepest part of the mass reaches near full thickness of the cervical muscle wall. The section appears a soft and delicate gray. (C) The tumor cells are arranged in a trabecular pattern and have an ovoid shape. The cells exhibit minimal cytoplasm (HE × 20). (D) CD56 positive. (E) Calretinin positive.

Except for the case of only diagnostic curettage, the remaining 6 tumors were macroscopically examined. The tumors ranged from 4 to 12 (mean = 8) cm. The majority of tumors were located in the intermuscular (4 cases), submucosal (1 case), and from the isthmus to external cervical os (1 case) (shown in Fig. 1). Five neoplasms were well-circumscribed and 1 had infiltrative borders. Tumor cells admixed with the smooth muscle component in these 7 cases. Lesions reached deep muscular layer in 3 cases but did not reach serous layer. Edema and degeneration were present in 2 cases, necrosis in 1 case. The cut surface had a fine, braided appearance and varied in color, with 4 out of 6 cases being grayish yellow, 1 off-white, and 1 gray pink. Microscopic examination revealed nests or cords, trabeculae, adenoid, reticular, and solid arrangements of tumor cells. The nuclear atypia of the tumor was not obvious, and the cell size was relatively consistent with less cytoplasm.

### 3.3. Immunohistochemical staining results

The tumor cells showed diffuse positivity for ER (7/7) and PR (4/7). All 7 tumors expressed 2 sex cord differentiation markers, CD56 (2/2), CD99 (4/5), calretinin (5/7), WT-1 (2/4), and α-Inhibin (1/7). Smooth muscle markers, including desmin (3/3), caldesmon (3/3), vimentin (2/2), and SMA (3/4), as well as the epithelial marker CK (4/7), were all expressed. However, no endometrial stromal marker CD10 was detected in any of the 3 cases. The Ki-67 proliferation index was <5% in 5 out of 7 cases and 20% in 2 out of 7 cases. CGA, syn, CD34, CD117, and Melan-A were all negative (shown in Fig. 1).

### 3.4. Follow-up

Post-surgical follow-up was conducted for all 7 patients, spanning 31 to 82 months (mean = 43 months). Six patients exhibited no evidence of recurrent or residual disease during follow-ups ranging from 31 to 82 months (mean = 41.3 months) postoperatively. Notably, 1 patient developed breast cancer 46 months post-surgery, a condition influenced by her family history; she received subsequent breast cancer surgery and chemotherapy. No recurrences of the primary uterine tumor were observed up to the last follow-up at 82 months. Another patient, who only received diagnostic curettage and no further surgical intervention, was followed up for 54 months; her death occurred with an undetermined cause.

## 4. Discussions

Since the initial report in 1976, Scully and Clement classified 2 types of tumors resemble each other histologically. There are significant differences in clinical behavior and genetic characteristics.^[1]^ Type II known as classical URTOSCT exhibit benign biological behavior, but also considered to be of low malignant potential. It has been reported in the literature that lymph node or even lung metastasis occurs,^[3,4]^ and serious deaths have been reported.^[5]^ The clinical characteristics are not fully understood, which poses challenges for preoperative diagnosis. This difficulty stems from its nonspecific clinical presentation, signs, and associated ancillary tests, leading to a low correct diagnosis rate. In order to shed light on this disease, we undertook a thorough examination of the clinicopathological features observed in 7 UTROSCT patients. This research involved a comprehensive review of relevant literature, enabling us to compile a summary and increase awareness of this disease.

### 4.1. Clinical manifestations and adjunctive tests

UTROSCT predominantly affects reproductive and perimenopausal women, with a median age of 49 years.^[6]^ The most common clinical manifestations include postmenopausal vaginal bleeding and menstrual irregularity, followed by pelvic pain.^[7]^ In some cases, UTROSCT may be asymptomatic and only incidentally detected.^[8]^ In line with previous reports,^[9]^ our study included patients aged between 38 and 66 years, with a mean age of 52 years, 3 were postmenopausal and 4 were perimenopausal women. The clinical presentation included menorrhagia being most common (5/7), followed by postmenopausal lower abdominal mass (2/7) and postmenopausal bleeding (1/7). Excessive menstruation and irregular vaginal bleeding induced secondary severe anemia (2/7).

Diagnosing UTROSCT based on medical imaging alone can be challenging, as it lacks significant diagnostic features. Currently, ultrasound examination is the most commonly used adjunctive test. Ultrasound findings may suggest an enlarged uterine corpus, intrauterine or intramural leiomyomas, or intrauterine polyps.^[8]^ In this study, gynecological ultrasound identified uterine fibroids in 4 cases (57.14%), adenomyosis combined with uterine fibroids in 2 cases (28.57%), and an intrauterine mass in 1 case (14.29%).

Pelvic MRI performed that the uterine cavity was enlarged, and the equal and high signals were filled with the uterine cavity.^[9]^ Two cases performed pelvic MRI in our study, with the suspicion of degeneration in uterine fibroids. In 1 case, the MRI revealed a solid cystic tumor shadow in the anterior wall of the uterus, raising suspicion of malignant transformation. Sarcomatous transformation could not be entirely ruled out. However, intraoperative findings revealed soft, yellowish tumors in the uterine cavity. The frozen pathological report tended to indicate endometrial stromal tumors, but postoperative pathology confirmed UTROSCT. Another patient, a 38-year-old woman with a preoperative diagnosis of submucosal fibroid, underwent hysteroscopic myomectomy. On frozen pathology examination, the excised tissue was revealed to be a uterine mesenchymal tumor with abundant cells forming nests. The pathology confirmed UTROSCT postoperatively. And then pelvic MRI was also performed on this patient, revealing clumps (or clots) in the uterine cavity base. However, this was attributed to postoperative changes. The patient subsequently underwent laparoscopic total hysterectomy with bilateral salpingectomy. The postoperative pathology showed partial loss of the endometrium and evidence of bleeding and inflammatory cell infiltration at the local basal and myometrial junction. These findings were consistent with postoperative changes, and no tumor residue was observed. The cases presented above highlight that compared to ultrasound, MRI can provide more precise information regarding tumor location and size. Additionally, MRI can be clinically valuable in determining the nature of the tumor. Clinicians should ensure thorough preoperative auxiliary examinations to guide treatment planning. MRI can also serve as an indicator to evaluate tumor lesion survival and is suitable for follow-up monitoring of patients who have undergone conservative treatment.

No sensitive serum tumor marker for UTROSCT has been identified thus far. In a study mentioned in the literature,^[10]^ a 49-year-old woman presented with a large uterine corpus measuring 13.9 × 9.4 × 12.2 cm, and the tumor marker CA125 was significantly elevated at 806.3 U/mL. However, CA199, CA15-3, AFP, and HE4 tumor markers were within normal limits. The patient subsequently underwent a total hysterectomy with bilateral adnexectomy, and the pathology confirmed UTROSCT. The post-surgery follow-up indicated an excellent prognosis. In this study, preoperative tumor marker tests were conducted on 6 patients, including CA125, CA199, CEA, AFP, and HE4. The laboratory results showed normal ranges in 5 patients, although 1 patient presented with an elevated CA125 marker of up to 158 U/mL. The correlation between the diagnosis of UTROSCT and the tumor marker CA125 should be focused on in the future.

### 4.2. Histopathological and immunohistochemical features

The pathological diagnosis remains the only definitive way to confirm the diagnosis in UTROSCT, and immunohistochemistry is used to aid in the differential diagnosis. The gross morphology of UTROSCT is typically located in the intermyometrial space, although a few cases have been found to be submucosal,^[11]^ and there have been rare instances reported in the uterine cervix.^[12,13]^ The tumor is well-defined, often nonencapsulated, and typically nodular in appearance. Macroscopically, the cut surface exhibits a homogeneous texture resembling fish flesh, with colors ranging from grayish yellow to grayish white and yellow. The tumor is predominantly solid or cystic in consistency. Microscopically, the morphology of the tumor cells closely resembles the sex cord tumor cells found in the ovaries, with a variable composition of sex cord components. The tumor cells arrange themselves in hollow tubules, cords, trabeculae, or sheets. Glandular structures may also be present. The cytoplasm is scant, and the nuclei are small and round with unremarkable nucleoli. Rare mitotic figures can be observed. UTROSCT is a multi-immunophenotypic tumor that expresses markers of epithelial, smooth muscle, and sex cord differentiation, as well as hormone receptor markers. Typically, more than 2 sex cord-stromal markers are expressed.^[6]^ According to the Irving study,^[14]^ calretinin exhibits 100% expression and is the sex cord marker with the highest concordance of expression in UTROSCT, while CD99 is expressed in 86% of cases (24/28). In this study, the morphology and arrangement of the tumor cells were consistent with UTROSCT. The immunohistochemistry results revealed that all cells expressed at least 2 sex cord differentiation markers, including CD56 (2/2), CD99 (4/5), calretinin (5/7), WT-1 (2/4), and inhibin (1/7). Additionally, smooth muscle markers such as desmin (3/3), caldesmon (3/3), vimentin (2/2), and SMA (3/4) were also expressed. Epithelial marker CK (4/7) was positive, while the endometrial stromal marker CD10 (0/3) was negative. The Ki-67 proliferation index was found to be <5% in 5 out of 7 cases and 20% in 2 cases, indicating relatively stable tumor cells. The findings demonstrate the significant role of immunohistochemistry in the diagnosis and differential diagnosis of UTROSCT.

In recent years, there have been significant advancements in molecular biological detection technology, making molecular testing a more promising method for diagnosing UTROSCT. Studies have found multiple NCOA, ESR1, or GREB1 gene fusions through RNA sequencing in patients with UTROSCT.^[15,16]^ Patients in the GREB1 rearranged group tended to be older and had larger tumor sizes and higher stages. Additionally, the tumors in the GREB1 rearranged group were predominantly intramural, whereas those in the non-GREB1 rearranged group were mostly polypoid/ submucosal tumors. It has been observed that tumors with GREB1::NCOA2 fusions are more likely to recur, indicating an aggressive and recurrent-prone biological behavior for this subtype of UTROSCT.^[15]^ Therefore, it is crucial to accurately diagnose UTROSCT and detect corresponding molecular alterations in order to predict the prognosis. Unfortunately, relevant molecular testing was not conducted in this cohort but was included when available.

### 4.3. Treatment modalities and prognosis

Most UTROSCTs demonstrate a benign behavior, as indicated by studies reporting no tumor recurrence or metastasis after total hysterectomy or oncologic resection with a maximum follow-up period of 12 years.^[11]^ However, in 2017, Moore^[4]^ reported a postoperative recurrence rate of up to 23.5% (8/34) and a mortality rate of 8.8% (3/34) in 34 patients with long-term follow-up. Further analysis revealed several high-risk factors associated with UTROSCT, including older patient age, large tumor size, tumor necrosis, lymphatic invasion, cervical involvement, significant nuclear atypia, and prominent karyorrhexis, with statistically significant differences found in both tumor necrosis and karyorrhexis. In 2018, a case of postoperative lung metastasis was reported in the literature.^[3]^ Hence, UTROSCT is still recognized as a tumor of uncertain malignant potential. In 2023, a series study by Baris^[17]^ of 75 cases emphasizing features predicting adverse outcome and differential diagnosis. Five of 58 patients with follow-up (22–192; mean = 73.2 months) had recurrences/metastases from 30 to 144 months, and 2 died of disease. Compared with benign tumors, malignant tumors showed more than 3 of the following 5 features: size > 5 cm, at least moderate cytologic atypia, >3 mitoses/10 HPF, infiltrative borders, and necrosis. The features listed above predict the aggressive behavior of this tumor.

Currently, total hysterectomy is considered the standard surgical approach for UTROSCT, with no impact on patient outcome observed from simultaneous removal of bilateral adnexa.^[18]^ However, for women with childbearing needs, conservative treatment modalities, such as resection of the uterine mass and close postoperative follow-up, can be chosen. Once childbearing is completed, resection of the uterus is still recommended. In 2021, a study by Carbone et al reported successful pregnancy and delivery have been reported in 2 patients treated conservatively.^[6]^ In this study, a 38-year-old patient with no fertility needs underwent hysteroscopic resection of a uterine cavity mass, followed by laparoscopic total hysterectomy and bilateral salpingectomy. Postoperative pathology revealed no tumor residue in the myometrial wall. Therefore, surgical complete removal of the lesion is essential for patient outcomes when conservative treatment is pursued. The cause of death, who only underwent diagnostic curettage was undetermined. This patient was followed for 54 months after the initial episode. The remaining 6 patients, who had their uteri resected, did not show any tumor recurrence or metastasis after surgery.

## 5. Conclusions

In conclusion, UTROSCT are rare, heterogeneous mesenchymal neoplasms of the uterus. They share similar morphological features to ovarian sex cord tumors, and histopathological examination after tumor resection is essential to definitively diagnose UTROSCT. Immunohistochemistry revealed at least 2 sex cord markers as positive but showed no recognizable endometrial stromal component. Therefore, determining the preoperative diagnosis of UTROSCT solely based on clinical examination and imaging studies is challenging. The biological behavior of UTROSCT remains uncertain, with the majority of cases being benign and a small proportion exhibiting low malignant potential. For patients not interested in preserving fertility, total hysterectomy is recommended. Alternatively, fertility-sparing surgery may be an option for patients who desire to conceive. Nonetheless, close follow-up is necessary until childbearing is complete, after which hysterectomy may be considered. MRI is more suitable for follow-up monitoring compared to ultrasound.

### 5.1. Future directions

To better understand and manage UTROSCT, future research should prioritize the development of diagnostic biomarkers, including the exploration of CA125 role in early detection and monitoring. Additionally, there is a crucial need to investigate the molecular pathology of UTROSCT, specifically the impact of gene fusions such as NCOA, ESR1, and GREB1 on tumor behavior and prognosis. The association of GREB1 fusions with more aggressive UTROSCT forms suggests these could be potential targets for therapeutic intervention and prognostic assessment.

## Acknowledgments

We appreciate the technical support provided by our hospital.

## Author contributions

**Conceptualization:** Zhe Ma.

**Data curation:** Ying Li, Zhe Ma.

**Investigation:** Zhe Ma.

**Methodology:** Zhe Ma.

**Resources:** Ying Li.

**Software:** Zhe Ma.

**Writing – original draft:** Zhe Ma.

**Writing – review & editing:** Zhe Ma.
